# The Genome Sequence of *Methanohalophilus mahii* SLP^T^ Reveals Differences in the Energy Metabolism among Members of the *Methanosarcinaceae* Inhabiting Freshwater and Saline Environments

**DOI:** 10.1155/2010/690737

**Published:** 2010-12-23

**Authors:** Stefan Spring, Carmen Scheuner, Alla Lapidus, Susan Lucas, Tijana Glavina Del Rio, Hope Tice, Alex Copeland, Jan-Fang Cheng, Feng Chen, Matt Nolan, Elizabeth Saunders, Sam Pitluck, Konstantinos Liolios, Natalia Ivanova, Konstantinos Mavromatis, Athanasios Lykidis, Amrita Pati, Amy Chen, Krishna Palaniappan, Miriam Land, Loren Hauser, Yun-Juan Chang, Cynthia D. Jeffries, Lynne Goodwin, John C. Detter, Thomas Brettin, Manfred Rohde, Markus Göker, Tanja Woyke, Jim Bristow, Jonathan A. Eisen, Victor Markowitz, Philip Hugenholtz, Nikos C. Kyrpides, Hans-Peter Klenk

**Affiliations:** ^1^DSMZ—German Collection of Microorganisms and Cell Cultures GmbH, 38124 Braunschweig, Germany; ^2^DOE Joint Genome Institute, Walnut Creek, CA 94598-1632, USA; ^3^Los Alamos National Laboratory, Bioscience Division, Los Alamos, NM 87545-001, USA; ^4^Biological Data Management and Technology Center, Lawrence Berkeley National Laboratory, Berkeley, CA 94720, USA; ^5^Oak Ridge National Laboratory, Oak Ridge, TN 37830-8026, USA; ^6^HZI—Helmholtz Centre for Infection Research, 38124 Braunschweig, Germany; ^7^Davis Genome Center, University of California, Davis, CA 95817, USA

## Abstract

*Methanohalophilus mahii* is the type species of the genus *Methanohalophilus*, which currently comprises three distinct species with validly published names. *Mhp. mahii* represents moderately halophilic methanogenic archaea with a strictly methylotrophic metabolism. The type strain SLP^T^ was isolated from hypersaline sediments collected from the southern arm of Great Salt Lake, Utah. Here we describe the features of this organism, together with the complete genome sequence and annotation. The 2,012,424 bp genome is a single replicon with 2032 protein-coding and 63 RNA genes and part of the *Genomic Encyclopedia of Bacteria and Archaea* project. A comparison of the reconstructed energy metabolism in the halophilic species *Mhp. mahii* with other representatives of the *Methanosarcinaceae* reveals some interesting differences to freshwater species.

## 1. Introduction

Halophilic methanogens contribute significantly to carbon mineralization in marine and hypersaline environments. The preferred substrates for methanogenesis in these habitats are C-1 methylated compounds, for example, methylamines that are constantly provided by the degradation of osmolytes like glycine betaine or membrane compounds such as choline. The preference of methylated C-1 compounds over hydrogen by methanogens thriving in saline environments reflects a competition with sulfate-reducing bacteria that are able to utilize hydrogen more efficiently than methanogens, but usually cannot use methanol or methylamines as substrates [1]. Strain SLP^T^ (= DSM 5219^T^ = ATCC 35705^T^) is the type strain of the moderately halophilic methanogen *Methanohalophilus mahii *[2]. SLP^T^ (Salt Lake Paterek) is the only strain of this species available from culture collections and was isolated from anoxic sediments of Great Salt Lake in Utah (USA) [3].

Although SLP^T^ represents the only described strain of this species, cultivation independent studies indicate that methanogens that are closely related to *Mhp. mahii* are quite common in anoxic saline environments. The 16S rRNA gene sequence of the nearest neighbor, *Methanohalophilus portucalensis* strain FDF-1, shares 99.8% sequence identity with SLP^T^ whereas the type strains of all other species in the *Methanosarcinales* share less than 94.7% with SLP^T^ [4]. Numerous cloned 16S rRNA genes more than 99% identical to the sequence of SLP^T^ were retrieved from hypersaline microbial mats of solar salterns in Guerrero Negro (Baja California Sur, Mexico) [5] and Eilat (Israel) [6], an endorheic hypersaline lake in La Macha, Spain (EF031086, unpublished), the deep-sea anoxic brine lake Urania, Eastern Mediterranean Sea (AM268272, unpublished) and an oilfield in Qinghai, China (EF190085, unpublished). In addition, clones of the methyl coenzyme M reductase alpha subunit (McrA) gene were obtained from hypersaline microbial mats in Guerrero Negro ponds (EU585971-EU585973, unpublished) that display high-sequence similarities (>97%) at the amino acid level with the McrA protein gene of *Mhp. mahii* (Mmah_0612). 

*Mhp. mahii *strain SLP^T^ is the first genome sequenced member of the genus *Methanohalophilus* representing moderately halophilic, methylotrophic methanogens. The comparison with the genomes of other freshwater and halophilic species belonging to the family *Methanosarcinaceae *provides novel insights into the genomic adaptations of methanogens to their environments.

## 2. Material and Methods

### 2.1. Growth Conditions and DNA Isolation

*Mhp. mahii *SLP^T^, DSM 5219^T^, was grown anaerobically in DSMZ medium 479 [7] at 37°C. DNA was isolated from 1–1.5 g of cell paste using MasterPure Gram Positive DNA Purification Kit (Epicentre MGP04100) according to the manufacturer's instructions with modification st/DLALM for cell lysis as described in Wu et al. 2009 [8].

### 2.2. Genome Project History

This organism was selected for sequencing on the basis of its phylogenetic position [9] and is part of the *Genomic Encyclopedia of Bacteria and Archaea* project [8]. The genome project is deposited in the Genomes OnLine Database [10] with the identifier Gc01255 and the complete genome sequence is accessible in GenBank as CP001994. Sequencing, finishing, and annotation were performed by the DOE Joint Genome Institute (JGI). A summary of the project information is shown in Table 1 in supplementary material available online at doi 10.1155/2010/690737.

### 2.3. Genome Sequencing and Assembly

The genome of *Mhp. mahii *SLP^T^ was sequenced using a 6.8 kb Sanger DNA library. All general aspects of library construction and sequencing performed at the JGI can be found at (http://www.jgi.doe.gov). Draft assemblies were based on 12,365 total reads. This library provided 6.8x coverage of the genome. The Phred/Phrap/Consed software package (http://www.phrap.com) was used for sequence assembly and quality assessment [11–13]. After the shotgun sequencing stage reads were assembled with parallel phrap (High Performance Software, LLC). Possible misassemblies were corrected with Dupfinisher [14]. Gaps between contigs were closed by editing in Consed, custom primer walks, or PCR amplification (Roche Applied Science, Indianapolis, IN). A total of 222 primer walk reactions and 5 pcr shatter libraries were necessary to close gaps, to resolve repetitive regions, and to raise the quality of the finished sequence. The completed genome sequence contains 14,767 reads, achieving an average of 8-fold sequence coverage per base with an error rate less than 1 in 100,000.

### 2.4. Genome Annotation

Genes were identified using Prodigal [15] as part of the Oak Ridge National Laboratory genome annotation pipeline, followed by a round of manual curation using the JGI GenePRIMP pipeline [16]. The predicted CDSs were translated and used to search the National Center for Biotechnology Information (NCBI) nonredundant database, UniProt, TIGRFam, Pfam, PRIAM, KEGG, COG, and InterPro databases. Additional gene prediction analysis and manual functional annotation was performed within the Integrated Microbial Genomes Expert Review (IMG-ER) platform [17].

### 2.5. Phylogenetic Analysis

Total genome protein sequences from 13 members of the class “*Methanomicrobia*” [18] and one outgroup taxon (*Haloterrigena turkmenica *[19], *Halobacteriaceae*) were retrieved from the IMG website (http://img.jgi.doe.gov/cgi-bin/geba/main.cgi) or from NCBI (*Methanocella paludicola* SANAE; NC_013665). “*Methanomicrobia”* appear as a sister group of *Halobacteriaceae* in a recent comprehensive 16S rRNA tree [20]. All-against-all protein BLAST was performed using mpiBLAST version 1.5 (http://www.mpiblast.org/), a parallel implementation of NCBI BLAST [21], using soft masking instead of complexity filtering. To determine orthologs, BLAST e-values were transformed using our own reimplementation of the OrthoMCL algorithm [22] in conjunction with MCL version 08-312 (http://micans.org/mcl/) using an inflation parameter of 2.0. OrthoMCL clusters containing inparalogs were reduced by selecting the most “central” of several sequences from the same genome, that is, the sequence with the highest sum of within-cluster BLAST scores. The reduced OrthoMCL clusters were aligned using MUSCLE version 3.7 [23]. The program scan_orphanerrs from the RASCAL package version 1.3.4 [24] was applied to detect orphan sequences within the alignments. After removal of orphan sequences (if present), poorly aligned columns and divergent regions were eliminated with GBLOCKS version 0.91 b [25] using a minimum block length of two amino acids and allowing gap positions in all sequences.

Filtered OrthoMCL cluster alignments comprising at least four sequences were concatenated to form a supermatrix for phylogenetic analysis. Maximum likelihood (ML) phylogenetic trees were inferred from the supermatrix with the Pthreads-parallelized RAxML package [26] version 7.2.5, applying fast bootstrapping with subsequent search for the best tree [27], the autoMRE bootstopping criterion [28] and the LG model of amino acid evolution [29] in conjunction with gamma-distributed substitution rates [30] and empirical amino acid frequencies. Tree searches under the maximum parsimony (MP) criterion were conducted with PAUP* version 4b10 [31] using 100 rounds of random sequence addition and subsequent TBR branch swapping, saving no more than 10 best trees per round and collapsing potential zero-length branches during tree search. MP bootstrap support was calculated with PAUP* using 1000 replicates with 10 rounds of heuristic search per replicate.

A comprehensive set of 16S rRNA sequences including most described species of *Methanosarcinales* were aligned using POA version 2.0 [32] and filtered with GBLOCKS. A phylogenetic tree was inferred under ML as described above but using the GTR+GAMMA substitution model.

## 3. Results and Discussion

### 3.1. Biology and Evolution of Mhp. mahii

#### 3.1.1. Phenotypic Characteristics

**Figure 1:**
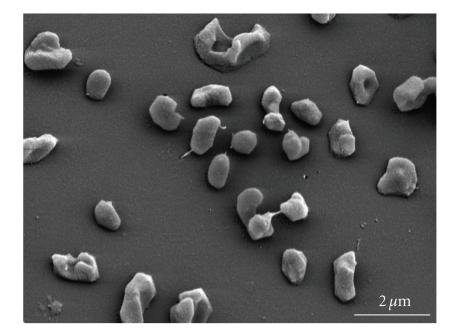
Scanning electron micrograph of cells of *Mhp. mahii *strain SLP^T^. Bar = 2 *μ*m.

Cells of *Mhp. mahii* SLP^T^ are irregular cocci with a diameter of 0.8 to 1.8 *μ*m that occur singly or in small clumps (Figure 1). Cells stain Gram-negative and are nonmotile [2]. Suitable salinities for growth of this moderately halophilic strain are in the range between 0.5 and 3.5 M NaCl. The highest growth rates are achieved in medium containing 2.0 M of sodium chloride, but the highest culture density was found at a salinity of 1.2 M NaCl [2]. Besides sodium, the metal ions Mg^2+^, K^+^, Ca^2+^, and Fe^2+^ are essential for methanogenesis and growth. The pH range for growth is 6.5 to 8.2 with an optimum at pH 7.5 [3].

*Mhp. mahii* SLP^T^ is strictly anaerobic and grows heterotrophically using methanol or methylamines as substrates for methanogenesis. No growth occurs lithotrophically with hydrogen and carbon dioxide or with the organic carbon sources acetate, formate, methionine, choline, or betaine [2]. Biotin stimulates growth of this strain in mineral medium with trimethylamine as substrate [33].

The polar lipid composition of *Mhp. mahii* strain SLP^T^ was analyzed by Koga et al. [34]. They identified the following lipid components: *β*-hydroxyarchaeol as core lipid, glucose as glycolipid sugar and *myo*-inositol, ethanolamine and glycerol as phospholipid-polar head groups. The deduced structure of the polar lipid diethers is in agreement with the current taxonomy that places the genus *Methanohalophilus* in the family *Methanosarcinaceae *[35]. Further data about chemotaxonomical traits in the genus *Methanohalophilus*, including the determination of cytochromes or methanophenazine are currently not available. The classification and general features of *Mhp. mahii* strain SLP^T^ in accordance with the MIGS recommendations [36] are summarized in Supplementary Table 2.

#### 3.1.2. Phylogeny and Taxonomy

According to current taxonomy halophilic methanogens are mainly affiliated to several genera within the family *Methanosarcinaceae.* Most representatives of this clade of halophilic archaea have a coccoid morphology and are methylotrophic and neutrophilic or alkaliphilic. Moderately halophilic methylotrophic species belong to the genera *Methanohalophilus* and *Methanosalsum*, slightly halophilic methanogens to the genera *Methanolobus* and *Methanococcoides* and extreme halophiles are classified in the genus *Methanohalobium*. In addition, the slightly halophilic species *Methanolobus siciliae* was described [37], but it was later transferred to the genus *Methanosarcina* [38]. At present the genus *Methanohalophilus* comprises three species with validly published names: *Mhp. mahii* (type species), *Mhp. halophilus* and *Mhp. portucalensis*. A fourth species, “*Mhp. euhalobius*”, was described by Obraztsova et al. [39] and Davidova et al. [40], but the species epithet has not been validated.

**Figure 2:**
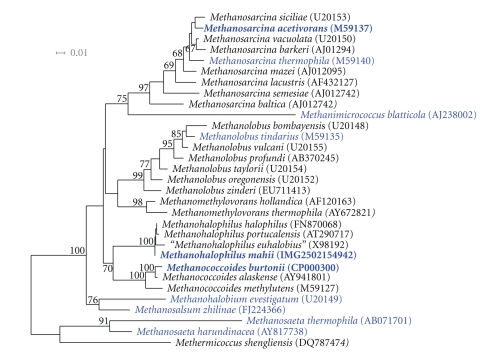
16S rRNA-based phylogenetic tree showing the position of *Mhp. mahii* relative to the other type strains within the order *Methanosarcinales*. The tree was inferred from 1341 aligned characters [25, 32] of the 16S rRNA gene sequence under the maximum likelihood criterion [26] and rooted with representatives of the *Methanocellaceae*. The branches are scaled in terms of the expected number of substitutions per site. Numbers above branches are support values from bootstrap replicates [28] if larger than 60%. Lineages with type strain-genome sequencing projects registered in GOLD [10] are shown in blue, published genomes in bold.

The 16S rRNA-based tree in Figure 2 shows the phylogenetic position of *Mhp. mahii *strain SLP^T^ among members of the order *Methanosarcinales*. The genome of strain SLP^T^ contains three copies of the 16S rRNA gene that differ by up to one nucleotide from the previously published 16S rRNA gene sequence generated from the same strain (M59133), which contains 23 ambiguous base calls. The difference between the genome data and the herereported 16S rRNA gene sequence is most likely due to sequencing errors in the previously reported sequence.

**Figure 3:**
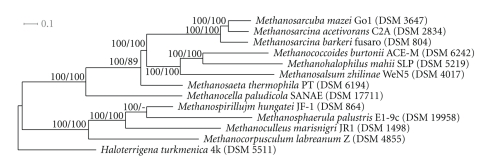
Maximum likelihood (ML) phylogenetic tree inferred from the 2344-gene supermatrix. The branches are scaled in terms of the expected number of substitutions per site. Numbers above branches are support values from ML (left) and maximum parsimony (MP; right) bootstrapping. The tree was rooted with the *Haloterrigena turkmenica* genome [19] included in the sample. The topology of the single best MP tree was identical to the one depicted here except for the unsupported branch connecting *Methanosphaerula palustris* and *Methanospirillum hungatei*.

In order to obtain a more accurate view on the evolution of halophilic members of the *Methanosarcinaceae* a phylogenetic reconstruction based on whole-genome data of sequenced representatives of the class “*Methanomicrobia” *[18] was performed. The number of aligned and filtered OrthoMCL clusters containing at least four entries (i.e., genes from distinct genomes) was 2344 and their length ranging from 27 to 1732 amino acids (267.45 on average). The concatenated supermatrix thus comprised 626,903 columns, including 577,297 variable and 291,638 parsimony-informative characters. The ML phylogeny inferred from the concatenated gene alignments is shown in Figure 3 together with ML and MP bootstrap support values. The final highest log likelihood obtained was −10,355,411.023, whereas the single best MP tree had a score of 1,907,449 (excluding uninformative sites). ML and MP topologies were identical except for a sister-group relationship between *Methanoculleus marisnigri* and *Methanosphaerula palustris*, which was preferred by MP (data not shown). Support was maximum (100%) for all branches under ML, and maximum for all but two branches under MP. The taxonomy was well recovered, and the tree showing the monophyly of all families and orders was included in the sample. However, the branching order within the family *Methanosarcinaceae* differed between the 16S rRNA- and whole-genome-based tree. Interestingly, the halophilic species *Methanococcoides burtonii*, *Methanosalsum zhilinae* and *Mhp. mahii*, which depend on NaCl for growth (Table 3), form a monophyletic branch in the whole-genome tree, whereas in the reconstructed 16S rRNA tree *Methanosalsum zhilinae *forms a separate line of descent together with the extremely halophilic species *Methanohalobium evestigatum*. Thus, it is likely that the tree based on genome-scale data is more accurate than the 16S rRNA tree and for this reason it is in higher agreement with the phenotype. In addition, the whole-genome-based tree indicates that in the evolution of methanogens production of cytochromes originated in the common ancestor of *Methanocellales *and *Methanosarcinales*. Originally, it was thought that the presence of cytochromes is restricted to the order *Methanosarcinales*, but recently genes encoding cytochromes were detected in methanogens from rice rhizosphere representing the newly proposed order *Methanocellales* [41].

### 3.2. Genome Structure and Content

**Figure 4:**
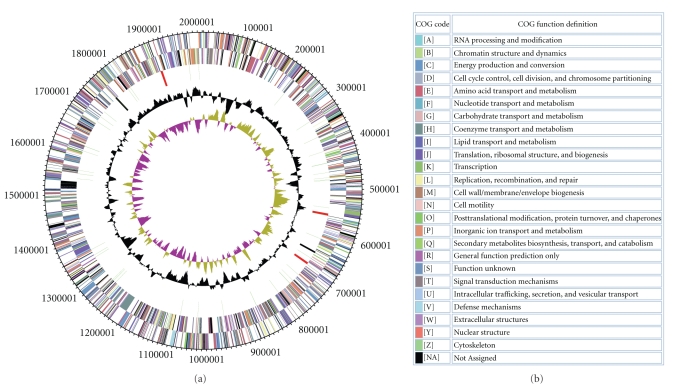
(a) Graphical circular map of the genome. From outside to the center: genes on forward strand (color by COG categories), genes on reverse strand (color by COG categories), RNA genes (tRNAs green, rRNAs red, and other RNAs black), GC content, GC skew. (b) Color code for COG categories.

The genome consists of a 2,012,424 bp long chromosome with a 42.6% GC content (Table 1 and Figure 4). Of the 2095 genes predicted, 2032 were protein-coding genes, and 63 RNAs; forty five pseudogenes were also identified. The majority of the protein-coding genes (69.7%) were assigned a putative function while the remaining ones were annotated as hypothetical proteins. The distribution of genes into COGs functional categories is presented in Table 2. 

### 3.3. Comparative Genome Analysis

Three different types of methanogens that can be mainly distinguished by the phylogenetic analyses of housekeeping genes and by some phenotypic traits have been proposed by Anderson et al. [44]. Class I methanogens belong to the orders *Methanobacteriales*, *Methanococcales* and *Methanopyrales*, class II methanogens are represented by the order *Methanomicrobiales* and class III methanogens by *Methanosarcinales*. The species *Mhp. mahii* belongs to the family *Methanosarcinaceae *and hence to the class III.

Postulated hallmark traits shared by all class III methanogens include the utilization of methyl compounds as sole substrate for methanogenesis, expression of cytochromes and the presence of genes encoding the following proteins: A, K, and N subunits of reduced coenzyme F_420_ (F_420_H_2_) dehydrogenase, a bacterial-type phosphoglycerate mutase, a bacterial adenylate kinase, phage shock protein A, the nonhistone chromosomal protein MC1 and an elongated variant of the condensin subunit ScpB [44]. The available genomic data set on representatives of the order *Methanosarcinales *is extended by presenting the complete genome of *Mhp. mahii* SLP^T^, thereby allowing a further evaluation of the postulated distribution of signature proteins in class III methanogens.

Furthermore, the adaptation of methanogenic pathways to increasing environmental salt concentration in members of the *Methanosarcinaceae* can be reconstructed by comparison of the genomes of the saltwater-adapted species *Mhp. mahii*, *Mco. burtonii* [45] and *Methanosarcina acetivorans* [46] with the genetic inventory of the freshwater preferring species *Msc. mazei* [47] and *Msc. barkeri* [48].

#### 3.3.1. Substrate Utilization and Methanogenesis

**Figure 5:**
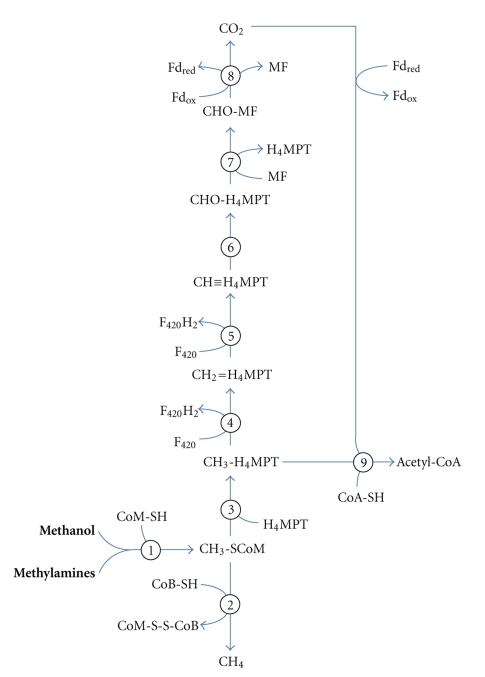
Proposed pathway of methanogenesis in *Mhp. mahii*. Numbers in circles indicate involved enzymes: (1), methyltransferase system; (2), methyl-CoM reductase; (3), methyl-H_4_MPT:HS-CoM methyltransferase; (4), methylene-H_4_MPT reductase; (5), methylene-H_4_MPT dehydrogenase; (6), methenyl-H_4_MPT cyclohydrolase; (7), formyl-MF:H_4_MPT formyltransferase; (8), formyl-MF dehydrogenase; (9), acetyl-CoA synthase/CO dehydrogenase. Abbreviations: Fd_red_, reduced ferredoxin; Fd_ox_, oxidized ferredoxin; MF, methanofuran; H_4_MPT, tetrahydromethanopterin; F_420_H_2_, reduced coenzyme  F_420_; F_420_, oxidized coenzyme F_420_; CoA-SH, coenzyme A; CoM-SH, coenzyme M; CoB-SH, coenzyme B; CoM-S-S-CoB, heterodisulfide of CoM and CoB.

Strain SLP^T^ is reported to grow exclusively on the C-1 compounds methanol and methyl amines whereas alternative substrates for methanogenesis like hydrogen, formate, and acetate are not utilized as substrates for growth [2]. A schematic drawing of the pathway of methanogenesis in *Mhp. mahii* is shown in Figure 5. The first step in methylotrophic methanogenesis is the transfer of a methyl group to coenzyme M (CoM). Then, the coenzyme M bound methyl group is reduced with coenzyme B (CoB) to methane, thereby leading to the formation of the CoM-S-S-CoB heterodisulfide. Methyltransferase systems of methanogens generally consist of the following three components that are localized in the cytoplasm: a methyl-accepting corrinoid protein (protein C), a substrate specific methyltransferase that catalyzes methylation of the corrinoid protein (protein B) and a methyltransferase that catalyzes methyl transfer from the methylated corrinoid protein to coenzyme M (MtbA). Methylation of the corrinoid protein requires reduction of the Co(II) corrinoid to the Co(I) state. The ATP-dependent reductive activation of the corrinoid protein is catalyzed in methylotrophic methanogens by the iron-sulfur protein RamA (Mmah_1683) [49]. In *Methanosarcina* species it was demonstrated that the corrinoid protein and the substrate specific methyltransferase form a tight complex and that the corresponding genes are transcribed in a single operon, whereas the MtbA genes are localized somewhere else in the genome and transcribed separately [50]. This seems also to be the case in the genome of *Mhp. mahii* strain SLP^T^, where methyltransferase systems for the following substrates were identified: methanol (Mmah_0975/Mmah_0976), monomethylamine (Mmah_1136/Mmah_1137 and Mmah_1154/Mmah_1155), dimethylamine (Mmah_0498/Mmah_0499 and Mmah_1674/Mmah_1675), and trimethylamine (Mmah_1680/1682). Genes encoding permeases for the uptake of methylamines could be identified in close proximity to the methyltransferase systems: Mmah_1133/1134 (monomethylamine transporter), Mmah_0500 (dimethylamine transporter) and Mmah_1679 (trimethylamine transporter). In addition, six different isozymes of methylcobalamin:coenzyme M methyltransferases (MtbA) could be detected: Mmah_0008, Mmah_0279, Mmah_0518, Mmah_0901, Mmah_0970, and Mmah_1478. In *Mhp. mahii* as in several other studied *Methanosarcina *species and* Mco. burtonii* genes encoding methylamine methyltransferases contain an amber codon (UAG), which causes normally a translation stop, but in this case can be recognized by a specific suppressor tRNA that carries the modified amino acid pyrrolysine [51]. It was shown that this amino acid plays an important role in the catalysis of the methyl transfer to the cognate corrinoid protein. Pyrrolysine is ligated to the amber decoding tRNA^Pyl^ by the pyrrolysyl-tRNA synthetase PylS (Mmah_0283).

The reduction of the CoM-bound methyl group to methane is catalyzed by the enzyme methyl-coenzyme M reductase (Mcr) that is exclusively found in methanogens. It is composed of the three subunits: McrA (Mmah_0612), McrB (Mmah_0616), and McrG (Mmah_0613) and contains the nickel porphinoid F_430_ as coenzyme. The reducing equivalents required for the reduction of the methyl group to methane are generated in strictly methylotrophic methanogens by the oxidation of the methyl group to carbon dioxide. Stoichiometrically, the oxidation of one methyl group to CO_2_ provides, electrons for the generation of three molecules of methane.

The first step in the oxidative branch of methylotrophic methanogenesis is the transfer of the methyl group from CoM to tetrahydromethanopterin (H_4_MPT), which is catalyzed by the membrane-bound enzyme complex tetrahydromethanopterin S-methyltransferase (MtrA-H). It has been shown that this multisubunit enzyme couples the exergonic transfer of the methylgroup from tetrahydromethanopterin to coenzyme M with the translocation of two sodium ions [52]. Thus, during methylotrophic methanogenesis this complex requires a sodium motive force to enable the endergonic transfer of the methylgroup from CoM to H_4_MPT. In *Mhp. mahii* all subunits of this enzyme are encoded in a single putative operon (Mmah_1776-1783), however, additional paralogs of some genes are found at several sites of the genome.

Subsequently, the enzymes methylene-H_4_MPT reductase (Mmah_1513) and methylene-H_4_MPT dehydrogenase (Mmah_0679) oxidize the methyl to a methenylgroup using the deazaflavin cofactor F_420 _as electron acceptor. The enzyme methenyl-H_4_MPT cyclohydrolase (Mmah_1138) then catalyzes the hydrolysis of methenyl-H_4_MPT to formyl-H_4_MPT. A further oxidation of the formyl group requires the transfer to the cofactor methanofuran (MF) by formyl-MF:H_4_MPT formyltransferase (Mmah_2027).

Finally, the large enzyme complex formyl-MF dehydrogenase oxidizes the formyl group to CO_2_ using a ferredoxin as a putative electron acceptor. It was previously shown that two different types of formyl-MF dehydrogenases are encoded in the genomes of *Methanosarcina *species, which is also observed in *Mhp. mahii*. Genes for a putative molybdate containing enzyme are arranged in a single unit (Mmah_1266–1271) whereas the presumably tungstate containing enzyme is encoded by genes that are localized at different sites or transcribed in different directions (Mmah_0590, Mmah_0950–952, Mmah_0956, and Mmah_1821). However, no gene encoding the subunit A of the tungstate containing formyl-MF dehydrogenase (*fwdA*) was identified, which may indicate that this enzyme is not functional in *Mhp. mahii*. Alternatively, the missing subunit could be replaced by the homologous subunit of the molybdate containing enzyme (FmdA).

Genes encoding enzymes for the utilization of alternative substrates for methanogenesis, like formate dehydrogenase (*fdhAB*), F_420_-dependent alcohol dehydrogenases (*adf*), or energy-conserving [NiFe] hydrogenases (*ech*) for the uptake of H_2_ were not detected in the annotated genome sequence, thus confirming the strict methylotrophy of this species. For the activation of acetate for methanogenesis *Methanosarcina *species use either acetate kinase (*ack*) and phosphotransacetylase (*pta*) or an ADP-forming acetyl-coenzyme A synthetase (*acdAB*). However, none of these genes were identified in the *Mhp. mahii* genome.

#### 3.3.2. Energy Metabolism

**Figure 6:**
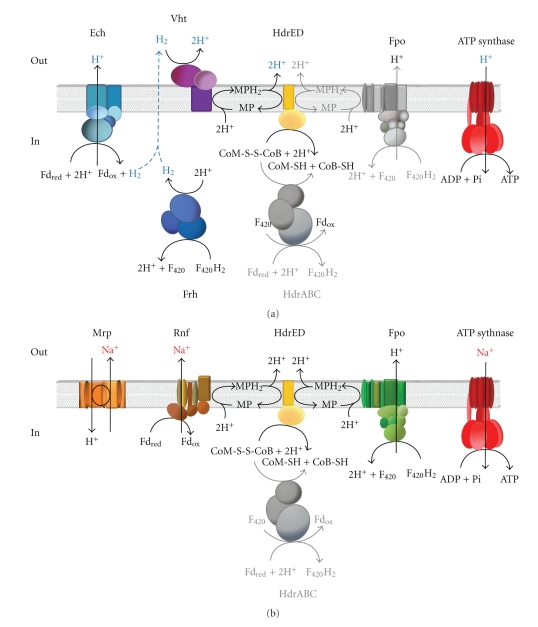
Comparison of proposed energy conserving electron-transfer routes in (a) freshwater-inhabiting and (b) saltwater-adapted members of the family *Methanosarcinaceae*. The proposed models of the energy metabolism are mainly based on the results of gene deletion and expression analyses in the species *Msc. barkeri* [54], *Msc. mazei* [55] and *Msc. acetivorans* [42, 56, 57]. The postulated coupling ion for ATP-synthesis is shown in blue (H^+^) or red (Na^+^). Enzyme complexes and reactions that are thought to bypass the main route of electron transfer are shown in grey. The shown electron bifurcation at the HdrABC complex is based on a proposal of Buan and Metcalf [58]. A membrane-bound sodium-translocating Mtr complex that could participate in the utilization of a chemiosmotic gradient was omitted from the figure for simplicity reasons. Abbreviations for electron transfer complexes and cofactors are explained in the text and Table 3.

The generation of metabolically useful energy in methylotrophic methanogens strictly depends on the buildup of a chemiosmotic gradient that can be utilized to form ATP by a membrane localized ATP synthase complex. The establishment of an ion-motive force usually requires membrane-bound protein complexes that convert the free energy change of electron transport processes in the translocation of protons or sodium ions outside of the cell. In methanogenic archaea, the energy-conserving electron transfer route is terminated by reduction of the heterodisulfide CoM-S-S-CoB to the corresponding thiol derivates CoM-SH and CoB-SH [41]. Possible electron donors for the electron transport chain in members of the *Methanosarcinaceae* depend on the substrate and can be molecular hydrogen, F_420_H_2 _or reduced ferredoxin [53]. A detailed description of the potential electron transfer routes and membrane-bound complexes involved in *Mhp. mahii*, including a comparison with the proposed pathways in* Methanosarcina *species, follows below and is illustrated in Figure 6.

**Hydrogenases and Hydrogen Cycling**

Recently, a hydrogen-cycling mechanism was postulated for the major mechanism of reducing the heterodisulfide reductase in *Msc. barkeri* [54] and *Msc. mazei* [55]. In these freshwater species the reduced cofactors ferredoxin (Fd_red_) and F_420_ (F_420_H_2_) are probably oxidized by the membrane-bound Ech hydrogenase and the cytoplasmic Frh F_420_-hydrogenase, respectively. The produced H_2_ is in turn oxidized by a membrane-bound methanophenazine-reducing hydrogenase (Vht/Vho). Finally, the reduced methanophenazine (MPH_2_) is used by the membrane-bound heterodisulfide reductase HdrED for reduction of CoM-S-S-CoB and concomitant proton translocation across the membrane [59] (Figure 6(a)). Interestingly, no genes encoding functional hydrogenases were identified in the *Mhp. mahii* genome. This would imply that H_2_ cannot be used for heterodisulfide reduction during methylotrophic growth. In Table 3 it is shown that genes encoding subunits of [NiFe] hydrogenase are also absent in *Mco. burtonii, *another halophilic species belonging to the *Methanosarcinaceae*. Although several types of hydrogenases are encoded in the halotolerant marine species *Msc. acetivorans*, experimental data have shown that no significant hydrogenase activity is present, so that H_2_ probably does not play a role as intermediate in methanogenic pathways of this species [56]. Thus, it is possible that the detected hydrogenase genes are silent or have an unknown function [42]. It is obvious from the above mentioned data that *Mhp. mahii* as well as *Msc. acetivorans* and *Mco. burtonii* must have evolved different strategies for the regeneration of reduced cofactors that do not rely on the generation of H_2_ as intermediate.

**Regeneration of F_420_**

In *Msc. mazei *and *Msc. barkeri,* an alternative route for the regeneration of F_420_ is possible that depends on the F_420_H_2_ dehydrogenase complex (Fpo) and indicates a branched electron transfer chain in these species. Fpo is a multimeric membrane-bound complex with proton translocation activity and has some similarities with the canonical NADH dehydrogenase I (Nuo) of aerobic bacteria [60]. In *Mhp. mahii* and other methanogens, this enzyme complex is encoded in a large putative operon (Mmah_1589–1601) with the conserved gene arrangement *fpoABCDHIJJKLMNO*. Only the gene of subunit FpoF (Mmah_1512) that contains the catalytic active site for F_420_H_2_ oxidation is encoded elsewhere. Interestingly, in the case of *Mhp. mahii,* it is adjacent to the gene of the methylene-H_4_MPT reductase (Mmah_1513), which represents one of the two enzymes in the oxidative branch of methylotrophic methanogenesis that reduce F_420_. Like the bacterial NADH dehydrogenase the Fpo F_420_H_2_ dehydrogenase of methanogens reduces a lipophilic redox carrier that is localized in the cytoplasmic membrane. Methanophenazine was identified in all hitherto studied species of the* Methanosarcinaceae* [41] as a functional analogue of the respiratory lipoquinones that are reduced by the bacterial NADH dehydrogenase. Thus, in *Mhp. mahii,* the Fpo dehydrogenase is probably responsible for the regeneration of the oxidized F_420_ cofactor using methanophenazine as electron acceptor. *Mhp. mahii *encodes also the membrane-bound heterodisulfide reductase HdrED (Mmah_0632/0633) that catalyzes the reduction of the heterodisulfide of CoM-S-S-CoB with MPH_2_ as reductant.

**Regeneration of Oxidized Ferredoxin**

Several mechanisms for the regeneration of oxidized ferredoxin without the generation of hydrogen have been recently proposed. A possible pathway involves a membrane-bound protein complex that was shown to be highly expressed in *Msc. acetivorans* and resembles the Rnf complex of bacteria [42]. It is assumed that in *Rhodobacter capsulatus,* this complex employs a chemiosmotic gradient to enable the endergonic reduction of ferredoxins with NADH [61]. In *Msc. acetivorans* electron transfer probably takes place in the reverse direction leading to the exergonic oxidation of ferredoxin with methanophenazine as electron acceptor and concomitant proton or sodium ion translocation [57]. Notably, freshwater species of *Methanosarcina* that encode an Ech hydrogenase catalyzing ferredoxin oxidation do not contain genes for a Rnf complex whereas in the genomes of *Mhp. mahii* (Mmah_1689–1696) and *Mco. burtonii* a putative Rnf operon was detected (Table 3), suggesting that this way of ferredoxin regeneration is the preferred option in halophilic and saltwater-inhabiting species of *Methanosarcinaceae* (Figure 6(b)).

An alternative route for ferredoxin oxidation was postulated by Buan and Metcalf [58]. Based on their findings obtained with gene-deletion mutants of *Msc. acetivorans,* they postulate that a cytoplasmic HdrABC complex uses reduced ferredoxin for the direct reduction of the CoM-S-S-CoB heterodisulfide. A possible advantage of this reaction would be that methanophenazine would not be required as an intermediate thereby allowing faster turnover rates for ferredoxin regeneration. Moreover, a possible growth-inhibiting overreduction of the methanophenazine pool by the Rnf complex is avoided. This assumption is supported by the finding that the cytoplasmic heterodisulfide reductase is especially important under conditions allowing fast growth of *Msc. acetivorans* in which regeneration of CoM-SH with reduced methanophenazine could represent a rate-limiting step [58]. In *Mhp. mahii* two sets of genes encoding cytoplasmic heterodisulfide reductases were found. One set is organized in a putative transcriptional unit (Mmah_0415–0417) whereas the other set encodes HdrA (Mmah_0955) and HdrBC (Mmah_1786/1787) separately.

**ATP Synthase Complex and Ion Motive Force**

A common genetic trait of halophilic representatives of *Methanosarcinaceae* seems to be the presence of genes encoding a multisubunit sodium/proton antiporter (Table 3). Antiporters of this type are encoded by six to seven different genes and were first described in bacteria as multiple-resistance/pH regulation (Mrp) complexes [62]. In *Mhp. mahii,* the Mrp complex is encoded in a single putative operon (Mmah_0736–0742). It has been proposed that these membrane-associated complexes function as proton-driven pumps for the efflux of sodium ions to achieve cellular homeostasis of pH and sodium [62]. Typically, genes encoding Mrp complexes are arranged as cassettes and often found in combination with distinct enzymes of the electron-transport chain, like formate dehydrogenases or energy converting hydrogenases that are supposed to translocate proton or sodium ions [63]. What could be the reason for the restricted distribution of the Mrp complex in halophilic species of the *Methanosarcinaceae*? One idea is that the Mrp complex plays a role in energy metabolism when chemiosmotic gradients of Na^+^ instead of protons are used for ATP synthesis. There is no direct evidence for this assumption, but it was shown by gene expression analyses that utilization of acetate in *Msc. acetivorans* stimulated production of the ATP synthase complex and the Mrp antiporter [42, 57], which might indicate that the Mrp complex is essential for the proper functioning of the ATP synthase. A tight coupling of electron transport with ATP synthesis is most important during the growth of methanogens on acetate, because the energy yield of this substrate is very small (ΔG^0′^ = −36 kJ/mol CH_4_) and close to the limit allowing cell growth [55]. A possible explanation for the absence of the Mrp complex in freshwater species of *Methanosarcina* is that their ATP synthases translocate protons instead of Na^+^,and hence are less dependent on sodium homeostasis compared to marine *Methanosarcina* species exposed to varying salinities and pH values.

In contrast to several *Methanosarcina* species that contain two different types of ATP synthases,* Mhp. mahii* contains only an ATP synthase of the archaeal A_1_A_0_-type that is encoded in a single putative operon (Mmah_1442–1450). Bioinformatic analyses of the ion-translocating proteolipid c-subunit of ATP synthases would indicate that Na^+^ translocation is used by all *Methanosarcina* species for the synthesis of ATP [42]. However, laboratory experiments of Pisa et al. [64] have shown that the ATP synthase of the freshwater species *Msc. mazei* depends on proton translocation, thus either bioinformatic predictions of the coupling ion are unreliable or ATP synthases of *Methanosarcina *species can depend on both, protons and sodium ions, depending on the growth conditions.

#### 3.3.3. Biosynthetic Metabolism

**Carbon Assimilation**

*Mhp. mahii* is able to grow in minimal media with methanol as sole carbon source, which indicates that all biosynthetic precursors can be synthesized from C-1 compounds. The first step of carbon assimilation is catalyzed by the cytoplasmic acetyl-CoA synthase/CO dehydrogenase complex (Mmah_1166–1172) that synthesizes acetyl-CoA from CO_2_, CoA, and methyl-H_4_MPT in a reaction that depends on reduced ferredoxin. Alternatively, external acetate may be transported into the cell and then activated via an adenosine 5′-monophosphate (AMP) and pyrophosphate forming acetyl-CoA synthase (Mmah_1375). The gene for a membrane-bound proton-translocating pyrophosphatase (Mmah_1380) is located in close proximity on the genome and may convert the chemical energy of the pyrophosphate bond into a chemiosmotic gradient that could be used for ATP synthesis, thereby regaining some of the energy invested for the activation of acetate. Carbon assimilation further proceeds by the reductive carboxylation of acetyl-CoA to pyruvate that is catalyzed by pyruvate:ferredoxin oxidoreductase (Mmah_0351/0354). The reduced ferredoxin required for carbon assimilation is probably provided by the formyl-MF dehydrogenase complex.

**Central Carbon Metabolism**

Starting from pyruvate, further carbon skeletons for biosynthesis can be either produced by the citric acid cycle or by gluconeogenesis. In *Mhp. Mahii,* the enzymes phosphoenolpyruvate synthase (Mmah_0997) and pyruvate phosphate dikinase (Mmah_0650) convert pyruvate into phosphoenolpyruvate (PEP) that can enter the gluconeogenic Embden-Meyerhof-Parnas (EMP) pathway by enolase (Mmah_1993) and phosphoglycerate mutase, which is present in an archaeal (Mmah_0223) and bacterial version (Mmah_1844), as in all studied members of the *Methanosarcinales *[44]. In *Mhp. mahii,* all necessary genes are present to operate the EMP pathway also in the glycolytic direction, which indicates that glycogen or starch is probably used as reserve material in this species and degraded under starvation conditions. On the other hand, pyruvate can be directed to the citric acid cycle by the enzyme pyruvate carboxylase (Mmah_0621/0622) that produces oxaloacetate. The important precursor metabolite succinyl-CoA is probably synthesized from oxaloacetate through the reductive branch of the citric acid cycle. Annotated genes representing enzymes of the reductive fork of the citric acid cycle include malate dehydrogenase (Mmah_1107), fumarase (Mmah_0724 and Mmah_1279/1280) and fumarate reductase (Mmah_0725/0726 and Mmah_1949/1950). Although genes for a canonical succinyl-CoA synthetase were not identified, it is possible that such an enzyme is encoded by the genes Mmah_1653 (potential ATPase) and Mmh_1654 (acyl-CoA synthetase), resulting in a complete set of enzymes for the partial reductive citric acid cycle. In *Mhp. mahii*, as in other members of the *Methanosarcinaceae* studied so far, 2-oxoglutarate is synthesized from oxaloacetate through the oxidative branch of the citric acid cycle using the enzymes citrate synthase (Mmah_0479), aconitase (Mmah_0478), and isocitrate dehydrogenase (Mmah_0582). It has to be considered that the citrate synthase gene of *Mhp. mahii* contains an amber stop codon at position 177 of the coding nucleotide sequence, so that it is currently designated as a pseudogene. On the other hand, 2-oxoglutarate is an indispensable precursor for the synthesis of glutamate and other essential amino acids, which leads to the assumption that the UAG stop codon could encode the noncanonical amino acid pyrrolysine as in the case of the methylamine methyltransferases discussed above.

**Cofactor Biosynthesis**

The enzymology of biochemical pathways for the synthesis of methanogenesis-specific cofactors has only recently begun to unveil. However, detailed studies on the synthesis of methanofuran and coenzyme F_430_ are still missing [65].

Coenzyme M biosynthesis was studied in *Methanocaldococcus jannaschii,* and a complete pathway including the involved genes was described [66]. Of the four enzymes catalyzing CoM synthesis in *Methanocaldococcus jannaschii* only the genes for sulfopyruvate decarboxylase (*comDE*; Mmah_1754/1755) were identified in the *Mhp. mahii* genome, which could indicate the existence of multiple pathways for sulfopyruvate production in methanogens. In contrast, several genes could be detected that probably encode enzymes participating in 2-oxoacid elongation steps required for synthesis of the 7-mercaptoheptanoyl chain of coenzyme B, that is, *aksA* (Mmah_1938), *aksD* (Mmah_0418), *aksE* (Mmah_0127), and *aksF* (Mmah_0129). Likewise, a complete set of genes (*cofC*, *cofD*, *cofE*, and *cofH*) involved in synthesis of the coenzyme F_420_ was identified in the *Mhp. mahii* genome.

All members of *Methanosarcinaceae* analyzed so far contain methanophenazine and cytochromes as part of their electron transport chains, so that genes encoding enzymes for their synthesis should be also present in *Mhp. mahii*. Only recently, Ogawa et al. [67] identified an enzyme in *Msc. mazei* that is specifically involved in the synthesis of methanophenazine. Methanophenazine contains a C-25 polyprenyl side-chain as lipophilic membrane anchor and an ether-linked redox-active phenazine moiety. It is thought that a specific step in the pathway of methanophenazine biosynthesis is the production of a C-25 prenyl group [(all-*E*) geranylfarnesyl diphosphate] that is subsequently combined with 2-hydroxyphenazine or its precursor. In *Msc. mazei* the synthesis of *all*-*E* geranylfarnesyl diphosphate is catalyzed by an (all-*E*) prenyl diphosphate synthase encoded by the gene MM_0789 whereas a homologous enzyme producing the C-20 (all-*E*) geranylgeranyl diphosphate is probably encoded by MM_1767 [67]. C-20 polyprenyl hydrocarbons are generally used in methanogenic archaea for the synthesis of membrane lipids, indicating that the distribution of this enzyme is not restricted to methanogens-producing methanophenazine. Indeed, it was found that highly similar sequences (*E* values <1*e* − 50) of the gene MM_0789 were found only in the genomes of *Mhp. mahii* (Mmah_1452) and other members of the *Methanosarcinales* whereas a similarly restricted distribution was not evident for the gene MM_1767. Interestingly, in the genome of *Mhp. mahii,* a cluster of genes required for heme biosynthesis (Mmah_1457–1461) and the ATP synthase operon are located in close proximity to the tentative (all-*E*) geranylfarnesyl diphosphate synthase gene (Mmah_1452), which could indicate a coordinated regulation of genes involved in electron transport phosphorylation.

#### 3.3.4. Protection against Osmotic and Oxidative Stress

**Halotolerance**

Halophilic or halotolerant microbes adapt to low water activity or varying salt concentrations by accumulating compatible solutes that balance the cell turgor under conditions of osmotic stress. The composition of osmolytes in *Mhp. mahii* is currently unknown but it was investigated in the phylogenetically related species *Mhp. portucalensis*. In this species the principal osmotic solutes are glycine betaine, *N* ^*ε*^-acetyl-*β*-lysine, *β*-glutamine and *α*-glucosylglycerate [68]. For energetic reasons the uptake of organic compatible solutes is preferred to *de novo* synthesis, hence transporters of common osmotic solutes should be encoded in the *Mhp. mahii* genome. Indeed, genes for an ABC-type transporter for glycine betaine (Mmah_0337–0340) were found. In addition, genes for alternative BetT-type choline transporters (Mmah_0372 and Mmah_1398) could be involved in the uptake of betaine or its precursors.

In *Methanosarcina* species transient osmotic stress is usually compensated by the intracellular potassium (K^+^) concentration [69]. Accordingly, in *Mhp. mahii* a K^+^ uptake system encoded by the genes *trkA* (Mmah_1614), *trkI* (Mmah_0056) and *trkH* (Mmah_2022) was identified and could play a role in a salinity-dependent increase of the cytoplasmic K^+^ concentration. Under conditions of hypoosmolarity efflux of K^+^ and Na^+^ ions could be catalyzed by KefC-type potassium/proton antiporters (Mmah_1757/Mmah_2010) and an NhaP-type sodium/proton antiporter (Mmah_1674), respectively. In addition, it is thought that mechanosensitive ion channels (*mscS1-3:* Mmah_0364, Mmah_0563, and Mmah_0666) that are gated by membrane tension play a role in the nonspecific efflux of solutes in response to hypoosmotic stress [70].

The immediate adaptation of cells to an increase of external salt concentrations by the rapid uptake of solutes is usually complemented by the cellular synthesis of organic osmolytes. In *Mhp. mahii,* the synthesis of *N* ^*ε*^-acetyl-*β*-lysine is catalyzed by lysine-2,3-aminomutase (*ablA*, Mmah_1439) and *β*-lysine acetyltransferase (*ablB*, Mmah_1438) whereas glycine betaine synthesis in methanogens relies on the genes for glycine sarcosine N-methyltransferase (Mmah_0524) and sarcosine dimethylglycine N-methyltransferase (Mmah_0525) [71].

**Oxygen Tolerance**

Cultivation-independent studies have revealed that methanogens closely related to the type strain of *Mhp. mahii* are abundant in the upper layers of photosynthetically active oxygenic microbial mats of solar salterns [5]. It is likely that these microbial mats are frequently exposed to UV light and oxygen, which lead to the generation of reactive oxygen species (ROS) being extremely toxic for obligately anaerobic cells. Consequently, members of this species must have acquired highly developed mechanisms to cope with oxidative stress. An analysis of the annotated genome sequence of *Mhp. mahii* indicates that the oxidative stress protection system in this species comprises multiple lines of defense characterized by enzymes reacting directly with oxygen and its derivatives or are involved in the repair of damaged cellular compounds. A whole array of enzymes is putatively involved in primary oxygen detoxification: Molecular oxygen entering the cell is reduced by a predicted F_420_H_2_ oxidase (*fprA,* Mmah_1537). The highly reactive superoxide radical (o_2_^−^) generated by auto-oxidation of thiols or flavins can be detoxified by two distinct desulfoferrodoxin-type superoxide reductases (Mmah_0553 and Mmah_1131) that reduce superoxide to hydrogen peroxide (H_2_O_2_). In turn, H_2_O_2_ is reduced to water by several types of peroxidases, including rubrerythrin or rubredoxin peroxidase (*rbr*, Mmah_0444), a ferritin-like Dps protein (*dps*, Mmah_0552), and a bacterial-type bifunctional catalase/peroxidase (*katG*, Mmah_1997).

On the other hand, an effective damage-repair mechanisms is installed that reacts with cellular compounds after they have become oxidized by ROS. *Mhp. mahii* encodes three different isozymes of 3-Cys thioredoxin peroxidases or peroxiredoxins (Mmah_0555, Mmah_1055, and Mmah_1299) that probably function as alkyl hydroperoxide reductases and repair oxidized lipids of the cytoplasmic membrane. Besides membrane lipids methionine residues of cytoplasmic proteins represent another prime target of ROS. The generation of methionine sulfoxide can lead to an alteration of the protein structure and other deleterious effects, so that it is necessary to reduce the sulfoxide moiety. In *Mhp. mahii,* the two different epimeric forms of methionine sulfoxide are reduced by the enzymes methionine-R-sulfoxide reductase (*msrB*, Mmah_0636) and methionine-S-sulfoxide reductase (*msrA*, Mmah_0990).

The electron transfer chain to desulfoferrodoxins, rubrerythrin, and peroxiredoxins is probably initiated by NADPH:thioredoxin reductase (Mmah_1201) or ferredoxin:thioredoxin reductase (Mmah_0953).

### 3.4. Conclusions

Anderson et al. proposed the classification of methanogens in three different classes based on a phylogenetic analysis of conserved genes and the distribution of signature proteins [44]. Based upon these criteria, *Mhp. mahii* is affiliated to class III methanogens except that no gene for the phage shock protein A was detected, which has been found so far in all members of the *Methanosarcinales*.

An interesting outcome of this study is that *Mhp. mahii* and other halophilic species of the *Methanosarcinaceae *use different energy-conserving electron routes during methylotrophic methanogenesis compared to freshwater species. *Msc. mazei *and *Msc. barkeri *rely on a hydrogen cycle with molecular H_2_ as intermediate whereas species adapted to saline environments prefer reduced cofactors for the intracellular transfer of reducing power. Obviously, the type of energy metabolism in *Methanosarcinaceae* does not correlate with the inherited halotolerance or the phylogenetic position, but seems to be the result of an adaptation to the occupied habitat, which explains why only *Msc. acetivorans* has omitted an H-cycle, but not the halotolerant freshwater species *Msc. mazei* and *Msc. barkeri*. It is tempting to speculate that the advantage of installing an H-cycle is the achievement of faster growth rates. Redox reactions of the rapidly diffusing H_2_ molecule can proceed very fast, because they do not rely on membrane-bound redox carriers that have to interact with large enzyme complexes, which is likely to slow down electron transfer. This would offer a possible explanation for the observation that methanogens without methanophenazines can have doubling times as low as one hour whereas methanogens with a membrane-bound eletron transport chain have generally doubling times above ten hours [41]. On the other hand, utilization of H_2_ as redox carrier could lead to the loss of reducing power from the cell, if freely diffusing H_2_ is scavenged by competing microorganisms. The abandonment of molecular hydrogen as intermediate substrate for methanogenesis in halophilic representatives of the *Methanosarcinaceae *can thus be explained by a competition with sulfate reducing microorganisms, which are usually only highly abundant in sulfate-rich saline environments and consume hydrogen more efficiently than methanogens [53, 72]. The observed effect of habitat salinity on the energy metabolism of methanogens seems to be restricted to members of the *Methanosarcinaceae*. This is probably based on the approximately tenfold lower hydrogen affinity of cytochrome-containing methanogens compared to methanogens of class I and class II, which are able to thrive by hydrogenotrophic methanogenesis in saline environments [41]. 

In summary, the obtained genome data allow the conclusion that the revealed abundance of *Mhp. mahii* in microbial mats of saline and hypersaline environments is based on a successful competition with other anaerobes, maybe by omitting molecular hydrogen as redox carrier, and by acquiring an oxidative stress protection system that is fully comparable to the system of microaerotolerant sulfate-reducing bacteria [73].

## Supplementary Material

Supplementary Table 1. Genome sequencing project information.
Supplementary Table 2. Classification and general features of *Mhp. mahii* strain SLP^T^ in accordance with the MIGS recommendations [36].
Evidence codes: TAS: Traceable Author Statement (i.e., a direct report exists in the literature); NAS: Non-traceable Author Statement (i.e., not directly observed for the living, isolated sample, but based on a generally accepted property for the species, or anecdotal evidence). These evidence codes are from the Gene Ontology project [74].
∗ In version 4.0 of the BMSB Taxonomic Outline [18] the class “*Methanomicrobia*” (including the orders *Methanomicrobiales* and *Methanosarcinales*) was created to replace the class *Methanococci*, whose definition seems to be in conflict with current analyses of complete genome data.

## Figures and Tables

**Table 1:** Genome Statistics.

Attribute	Value	% of Total
Genome size (bp)	2,012,424	100.00
DNA coding region (bp)	1,791,905	89.04
DNA G+C content (bp)	857,659	42.62
Number of replicons	1	
Extrachromosomal elements	0	
Total genes	2095	100.00
RNA genes	63	0.43
rRNA operons	3	
Protein-coding genes	2032	96.99
Pseudo genes	45	2.15
Genes with function prediction	1461	69.74
Genes inparalog clusters	265	12.65
Genes assigned to COGs	1596	76.18
Genes assigned Pfam domains	1629	77.76
Genes with signal peptides	204	9.74
Genes with transmembrane helices	431	20.57
CRISPR repeats	0	

**Table 2:** Number of genes associated with the general COG functional categories.

Code	COG counts and percentage of protein-coding genes		
	Genome		Description
	value	% of total	
J	158	9.3	Translation, ribosomal structure, and biogenesis
A	2	0.1	RNA processing, and modification
K	75	4.4	Transcription
L	84	5.0	Replication, recombination and repair
B	2	0.1	Chromatin structure and dynamics
D	17	1.0	Cell cycle control, cell division, and chromosome partitioning
Y	0	0.0	Nuclear structure
V	14	0.8	Defense mechanisms
T	72	4.3	Signal transduction mechanisms
M	41	2.4	Cell wall/membrane/envelope biogenesis
N	5	0.3	Cell motility
Z	0	0.0	Cytoskeleton
W	0	0.0	Extracellular structures
U	19	1.1	Intracellular trafficking and secretion, and vesicular transport
O	81	4.8	Posttranslational modification, protein turnover, and chaperones
C	153	9.0	Energy production and conversion
G	49	2.9	Carbohydrate transport and metabolism
E	147	8.7	Amino acid transport and metabolism
F	51	3.0	Nucleotide transport and metabolism
H	150	8.9	Coenzyme transport and metabolism
I	22	1.3	Lipid transport and metabolism
P	102	6.0	Inorganic ion transport and metabolism
Q	14	0.8	Secondary metabolites biosynthesis, transport and catabolism
R	228	13.5	General function prediction only
S	209	12.3	Function unknown
—	499	23.8	Not in COGs

**Table 3:** The distribution of genes involved in the energy metabolism of members of the *Methanosarcinaceae* shows a correlation with the occupied habitat. The designation of genes is based on the proposed scheme of Rohlin and Gunsalus [42]. Phenotypic characteristics of the listed species were taken from Bergey's Manual of Systematic Bacteriology [43]. Abbreviations: *Msc, Methanosarcina; Mco*, *Methanococcoides; Msl, Methanosalsum; Mhb, Methanohalobium; Mhp, Methanohalophilus;* +, genes present; −, genes not detected; (+), genes are present, but known to be silent. Note: at the time of writing, the genome sequence of *Methanohalobium evestigatum* (CP002069) was only available as draft.

	*Msc.* *barkeri *	*Msc.* *mazei *	*Msc.* *acetivorans*	*Mco.* *burtonii *	*Msl.* *zhilinae *	*Mhb.* *evestigatum *	*Mhp.* *mahii*
Growth conditions:							
Salinity of normal habitat	Freshwater	Freshwater	Marine	Marine	Hypersaline	Hypersaline	Hypersaline
NaCl requirement	−	−	+	+	+	+	+
NaCl optimum [M]	0.2	0.2	0.2	0.2	0.4– 0.7	4.3	1.2–2.0
Temp. optimum [°C]	35	35	37	23	45	50	35

Substrates:							
Acetate	+	+	+	−	−	−	−
methanol, methylamines	+	+	+	+	+	+	+
H_2_ + CO_2_	+	+	−	−	−	−	−

Presence of genes:							
Strain sequenced	Fusaro	Gö1	C2A^T^	ACE-M^T^	WeN5^T^	Z-7303^T^	SLP^T^
*vhoGAC *	−	+	−	−	−	−	−
*vhtGACD *	+	+	+	−		−	−
*vhtGADF *	−	−	−	−	+	−	−
*vhtGAC *	+	+	(+)	−	−	−	−
*frhADGB *	+	+	(+)	−	−	−	−
*echABCDEF *	+	+	−	−	−	−	−
*fpoABCDHIJJKLMNO *	+	+	+	+	+	+	+
*rnfXCDGEABY *	−	−	+	+	+	+	+
*mrpABCDEFG *	−	−	+	+	+	+	+
*hdrED *	+	+	+	+	+	+	+
*hdrACB *	+	+	+	+	+	+	+
*ahaHIKECFABD *	+	+	+	+	+	+	+
