# Supplementary material for: The Genome Sequence of Methanohalophilus mahii SLPT Reveals Differences in the Energy Metabolism among Members of the Methanosarcinaceae Inhabiting Freshwater and Saline Environments
Source: Archaea. 2010 Dec 23;2010:690737. doi: 10.1155/2010/690737 (PMC3017947; doi:10.1155/2010/690737)
Supplement: Supplementary file 1 — Supplementary Table 1. Genome sequencing project information. Supplementary Table 2. Classification and general features of Mhp. mahii strain SLPT in accordance with the MIGS recommendations [36]. Evidence codes: TAS: Traceable Author Statement (i.e., a direct report exists in the literature); NAS: Non-traceable Author Statement (i.e., not directly observed for the living, isolated sample, but based on a generally accepted property for the species, or anecdotal evidence). These evidence codes are from the Gene Ontology project [74]. ∗ In version 4.0 of the BMSB Taxonomic Outline [18] the class “Methanomicrobia” (including the orders Methanomicrobiales and Methanosarcinales) was created to replace the class Methanococci, whose definition seems to be in conflict with current analyses of complete genome data. [file 690737.f1.pdf]

Supplementary Material

Supplementary Table 1. Genome sequencing project information.

MIGS ID	Property	Term
MIGS-31	Finishing quality	Finished
MIGS-28	Libraries used	One genomic 6 kb pMCL Sanger library
MIGS-29	Sequencing platforms	ABI3730
MIGS-31.2	Sequencing coverage	8.0 Sanger
MIGS-30	Assemblers	phrap
MIGS-32	Gene calling method	Prodigal 1.4, GenePRIMP
	GenBank ID	CP001994
	GenBank Date of Release	April 12, 2010
	GOLD ID	Gc01255
	NCBI project ID	547558
	Database: IMG-GEBA	2502082111
MIGS-13	Source material identifier	DSM 5219^T
	Project relevance	GEBA

Supplementary Table 2. Classification and general features of *Mhp. mahii* strain SLP^T in accordance with the MIGS recommendations [36]. Evidence codes: TAS: Traceable Author Statement (i.e., a direct report exists in the literature); NAS: Non-traceable Author Statement (i.e., not directly observed for the living, isolated sample, but based on a generally accepted property for the species, or anecdotal evidence). These evidence codes are from the Gene Ontology project [74].

* In version 4.0 of the BMSB Taxonomic Outline [18] the class "*Methanomicrobia*" (including the orders *Methanomicrobiales* and *Methanosarcinales*) was created to replace the class *Methanococci*, whose definition seems to be in conflict with current analyses of complete genome data.

MIGS ID	Property	Term	Evidence code
		Domain *Archaea*	TAS [75]
		Phylum *Euryarchaeota*	TAS [76]
		Class *Methanococci**	TAS [73]
	Current classification	Order *Methanosarcinales*	TAS [76]
		Family *Methanosarcinaceae*	TAS [77]
		Genus *Methanohalophilus*	TAS [2]
		Species *Methanohalophilus mahii*	TAS [2]
		Type strain SLP^T	TAS [2]
	Gram stain	negative	TAS [2]
	Cell shape	coccoid	TAS [2]
	Motility	nonmotile	TAS [2]
	Sporulation	negative	TAS [2]
	Temperature range	20 – 45 °C	TAS [3]
	Optimum temperature	35°C	TAS [2]
	Salinity	0.5 M – 3.5 M NaCl	TAS [3]
	Optimum salinity	1.2 – 2.0 M NaCl	TAS [3]
MIGS-22	Oxygen requirement	obligate anaerobic	TAS [2]
	Carbon source	methanol, methylamine, dimethylamine, trimethylamine	TAS [2]
	Energy source	see above	TAS [2]
MIGS-6	Habitat	saline to hypersaline anoxic sediments	TAS [2]
MIGS-15	Biotic relationship	unknown	

MIGS-14	Pathogenicit y	none	TAS [78]
	Biosafety level	1	TAS [78]
	Isolation	water/sediment sample	TAS [2]
MIGS-4	Geographic location	Great Salt Lake (south arm), Utah, USA	TAS [3]
MIGS-5	Sample collection time	1985 or before	NAS
MIGS-4.1<br>MIGS-4.2	Latitude – Longitude	41.177 – -112.502	NAS
MIGS-4.3	Depth	not reported	
MIGS-4.4	Altitude	not reported	
